# Supplementary material for: CUL-6/cullin ubiquitin ligase-mediated degradation of HSP-90 by intestinal lysosomes promotes thermotolerance
Source: Cell Rep. Author manuscript; Available in PMC 2024 Jul 11. (PMC11238739; doi:10.1016/j.celrep.2024.114279)
Supplement: 1 [file NIHMS2005217-supplement-1.pdf]

Cell Reports, Volume *43*

Supplemental information

CUL-6/cullin ubiquitin ligase-mediated degradation of HSP-90 by intestinal lysosomes promotes thermotolerance

Mario Bardan Sarmiento, Spencer S. Gang, Patricija van Oosten-Hawle, and Emily R. Troemel

Figure S1

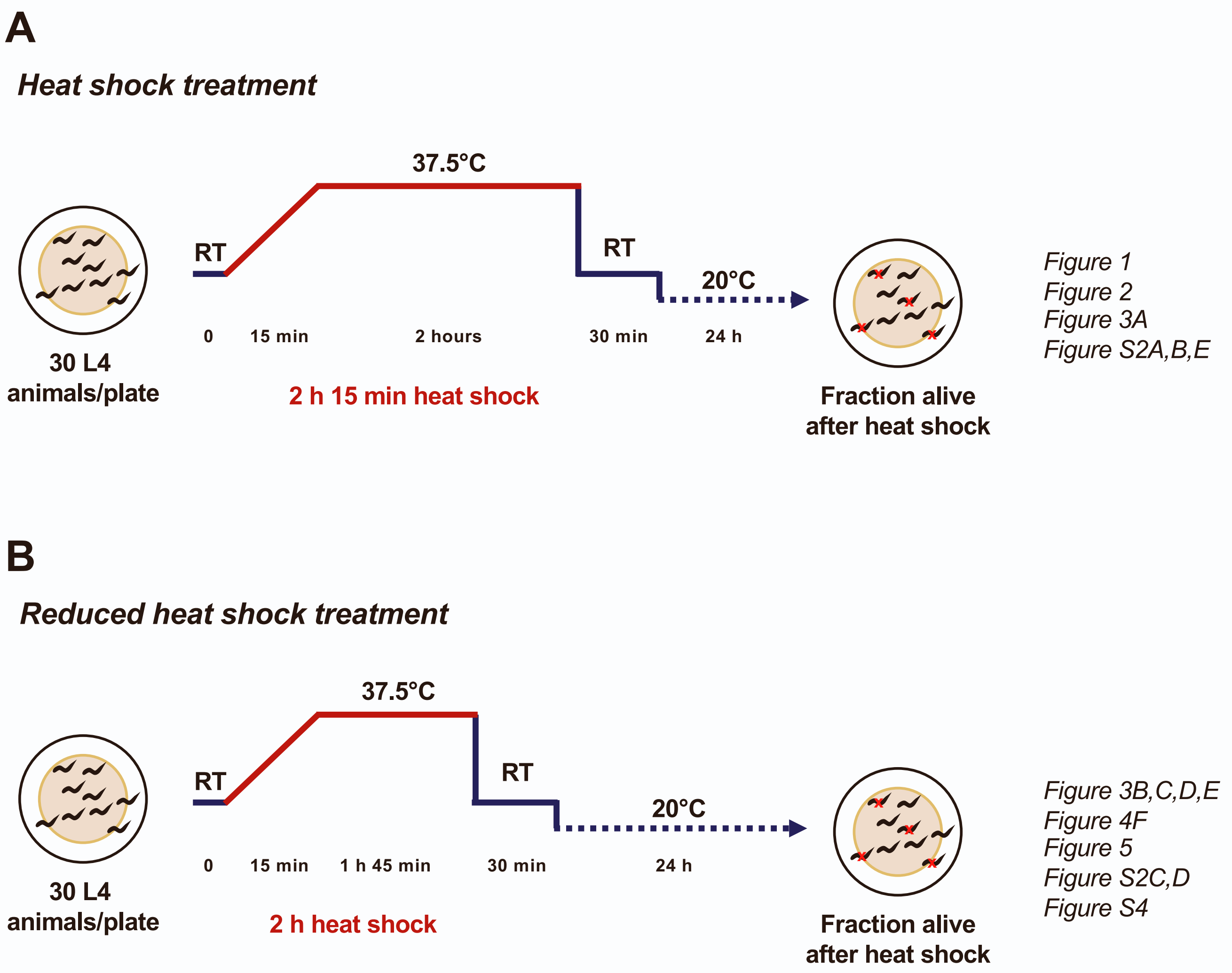
A
Heat shock treatment
37.5°C
RT
RT
20°C
0
15 min
2 hours
30 min
24 h
30 L4
animals/plate
2 h 15 min heat shock
Fraction alive
after heat shock
Figure 1
Figure 2
Figure 3A
Figure S2A,B,E
B
Reduced heat shock treatment
37.5°C
RT
RT
20°C
0
15 min
1 h 45 min
30 min
24 h
30 L4
animals/plate
2 h heat shock
Fraction alive
after heat shock
Figure 3B,C,D,E
Figure 4F
Figure 5
Figure S2C,D
Figure S4

Figure S1. Heat shock treatments to assess thermotolerance phenotypes. Related to All Figures (specified in panels A and B).

(A) Diagram of the workflow for the 2 h 15 min “heat shock treatment” and experiments where it was applied (also see methods). (B) Diagram of the workflow for the 2 h “reduced heat shock treatment” and experiments where it was applied (also see methods). HSP-90$^{int\text{-}OE}$ strains survive poorly following the longer heat shock treatment shown in A (example: Figure 3A, control condition). When warranted, strains with HSP-90$^{int\text{-}OE}$ were instead subjected to the less stressful reduced heat shock treatment to better assess CUL-6 and lysosome-mediated phenotypes.

Figure S2

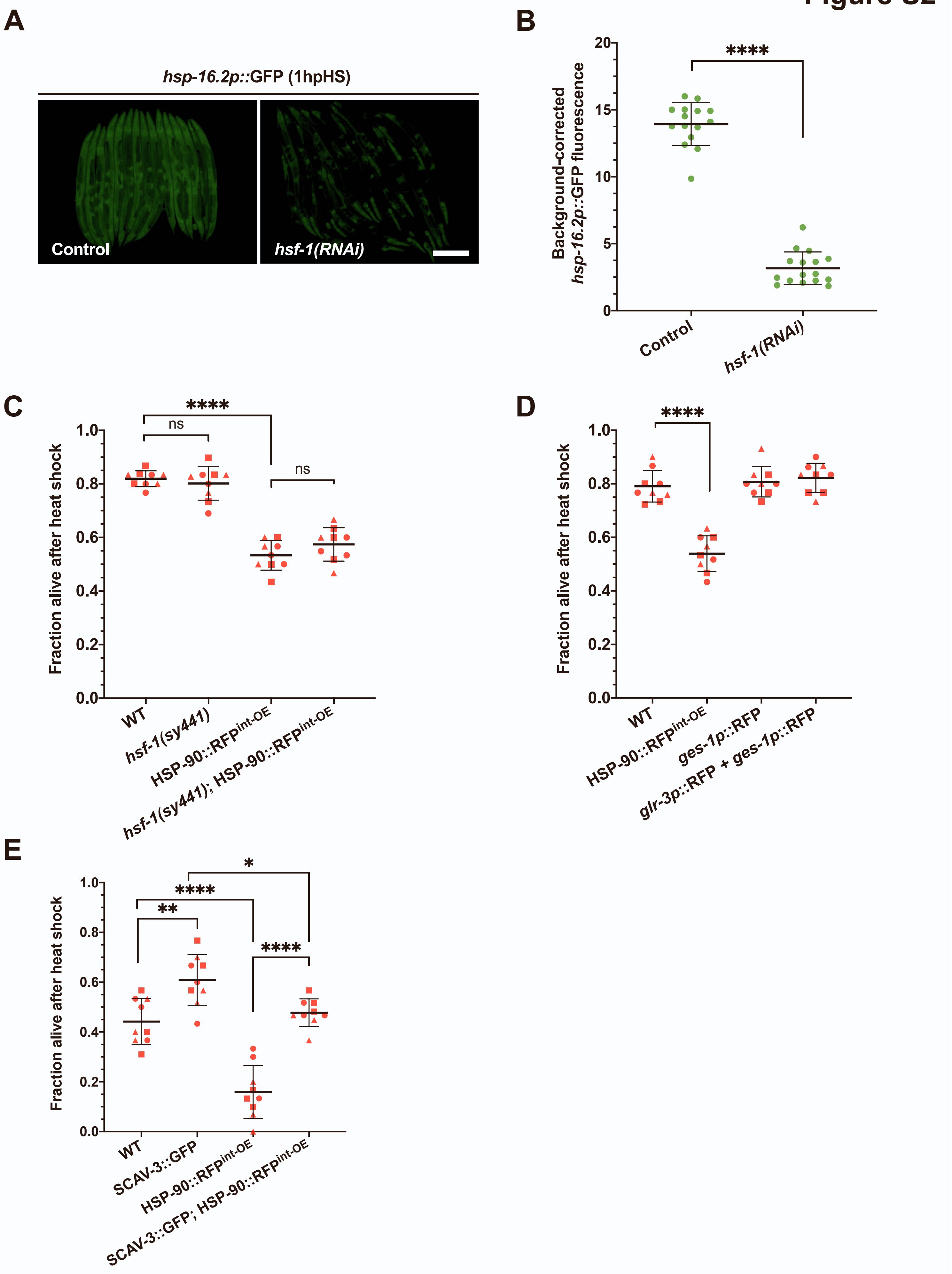
A
hsp-16.2p::GFP (1hpHS)
Control
hsf-1(RNAi)
B
Background-corrected
hsp-16.2p::GFP fluorescence
****
20
15
10
5
0
Control
hsf-1(RNAi)
C
Fraction alive after heat shock
****
ns
ns
1.0
0.8
0.6
0.4
0.2
0.0
WT
hsf-1(sy441)
HSP-90::RFPint-OE
hsf-1(sy441); HSP-90::RFPint-OE
D
Fraction alive after heat shock
****
WT
HSP-90::RFPint-OE
ges-1p::RFP
glr-3p::RFP + ges-1p::RFP
E
Fraction alive after heat shock
*
****
**
****
WT
SCAV-3::GFP
HSP-90::RFPint-OE
SCAV-3::GFP; HSP-90::RFPint-OE

Figure S2. CUL-6 lowers HSP-90::RFP levels independent of HSF-1's heat shock-inducible functions, and overexpression of SCAV-3::GFP promotes thermotolerance

(A) Fluorescent images of L4 animals showing induction of *hsp-16.2p::*GFP 1 h post heat shock after RNAi against *hsf-1* relative to control RNAi. Scale bar = 200 µm. (B) Quantification of *hsp-16.2p::*GFP signal of animals shown in panel A. An unpaired t-test was used to calculate the p-value. (C) Survival of wild-type or *hsf-1(sy441)* mutants without or with HSP-$90^{int\text{-}OE}$, and HSP-$90^{int\text{-}OE}$ animals after reduced heat shock treatment. (D) Survival of wild-type, HSP-$90^{int\text{-}OE}$ animals, and two additional strains that overexpress RFP in the cytosol of the intestine after reduced heat shock treatment. (E) Survival of wild-type and HSP-$90^{int\text{-}OE}$ animals with or without SCAV-3::GFP overexpression after heat shock treatment. For C-E, animals were tested in triplicate experiments with three plates per experiment and 30 animals per plate. The mean fraction of animals alive for the pooled replicates is indicated by the black bar with error bars as the SD. Each dot represents a plate, and different shapes represent the experimental replicates performed on different days. A one-way ANOVA with Tukey's multiple comparisons test was used to calculate p-values.; ****$p <0.0001$; **$p < 0.01$; *$p < 0.05$.

Figure S3

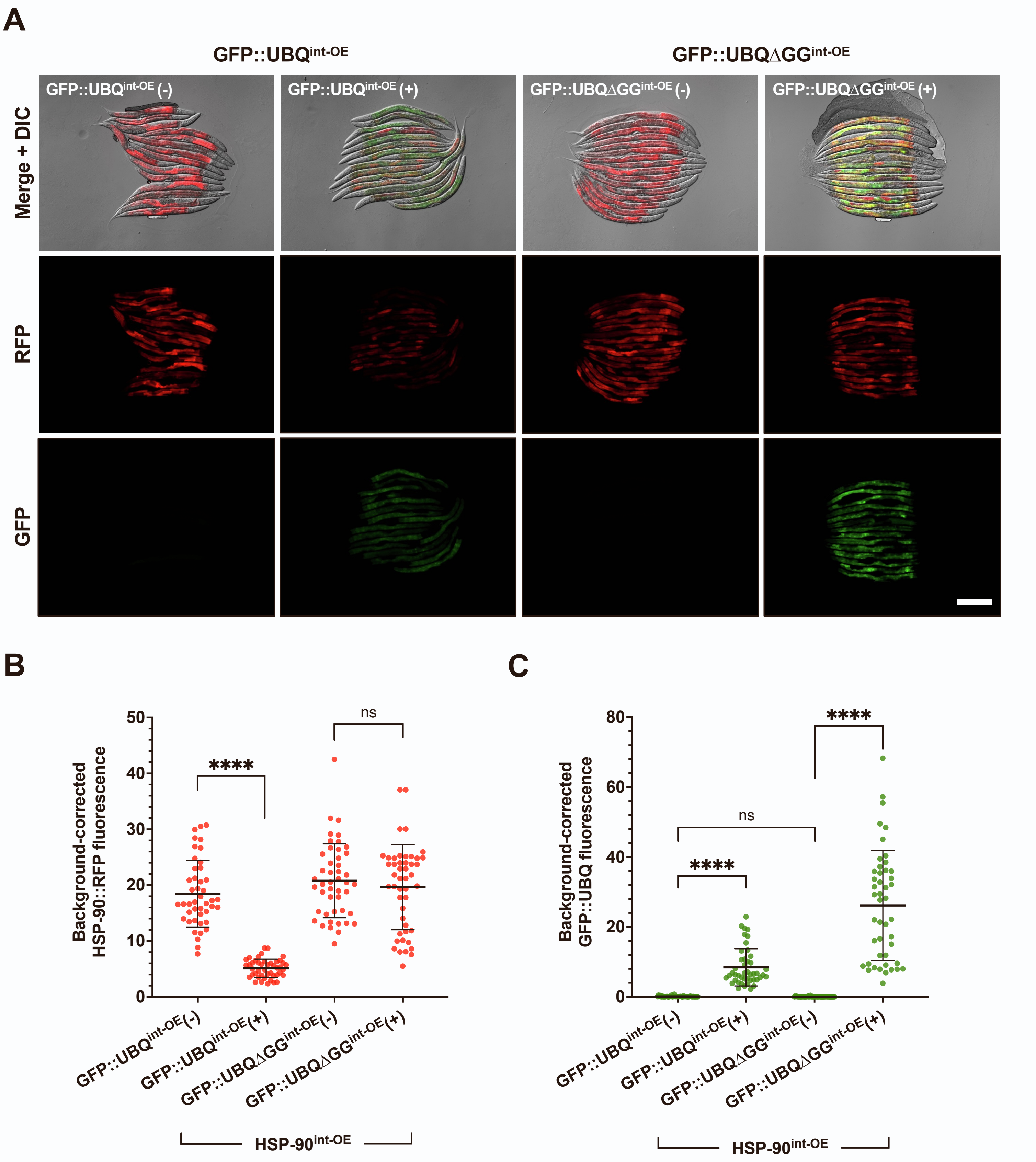
A
GFP::UBQ^int-OE
GFP::UBQΔGG^int-OE
GFP::UBQ^int-OE (-)
GFP::UBQ^int-OE (+)
GFP::UBQΔGG^int-OE (-)
GFP::UBQΔGG^int-OE (+)
Merge + DIC
RFP
GFP
B
Background-corrected HSP-90::RFP fluorescence
50
40
30
20
10
0
****
ns
GFP::UBQ^int-OE(-)
GFP::UBQ^int-OE(+)
GFP::UBQΔGG^int-OE(-)
GFP::UBQΔGG^int-OE(+)
HSP-90^int-OE
C
Background-corrected GFP::UBQ fluorescence
80
60
40
20
0
****
ns
****
GFP::UBQ^int-OE(-)
GFP::UBQ^int-OE(+)
GFP::UBQΔGG^int-OE(-)
GFP::UBQΔGG^int-OE(+)
HSP-90^int-OE

Figure S3. Ubiquitin overexpression in the intestine reduces HSP-90::RFP expression

(A) Representative fluorescent images of L4 animals carrying an extrachromosomal array that intestinally overexpresses either functional GFP::UBQ or a conjugation-deficient version, GFP::UBQΔGG, in an HSP-$90^{int\text{-}OE}$ background. Sibling transgenic animals carrying both the GFP::UBQ/GFP::UBQΔGG array and HSP-$90^{int\text{-}OE}$ or just HSP-$90^{int\text{-}OE}$ alone are shown. Scale bar = 200 μm. (B) Quantification of the RFP fluorescent signal for each condition. (C) Quantification of the GFP fluorescent signal for the same animals measured in B. For B and C, each dot represents one animal measured, and the data shown are the results of three experimental replicates. n = 44 – 47 worms quantified. The black bar indicates the mean fluorescence intensity with error bars as SD. For each, a Kruskal-Wallis test was used to calculate p-values; ****p <0.0001.

Figure S4

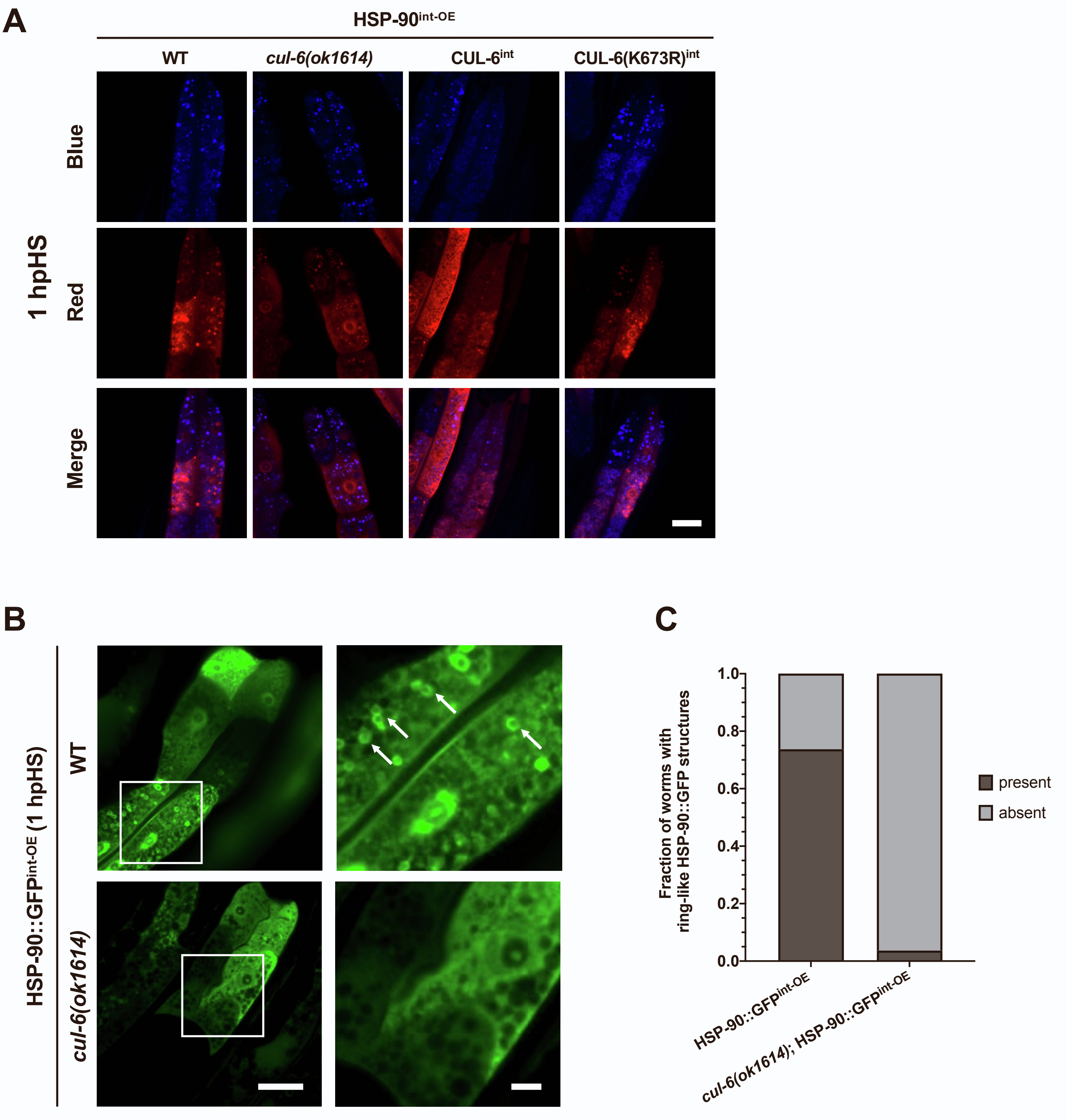

A
HSP-90int-OE
WT
cul-6(ok1614)
CUL-6int
CUL-6(K673R)int
1 hpHS
Blue
Red
Merge
B
HSP-90::GFPint-OE (1 hpHS)
WT
cul-6(ok1614)
C
Fraction of worms with ring-like HSP-90::GFP structures
1.0
0.8
0.6
0.4
0.2
0.0
present
absent
HSP-90::GFPint-OE
cul-6(ok1614); HSP-90::GFPint-OE

Figure S4. Ring-like structures formed after heat shock appear to be HSP-90-specific

(A) Confocal fluorescence images split by channel of the anterior intestine in L4 animals with varying levels of CUL-6 expression in strains in an HSP-90$^{int\text{-}OE}$ background 1 h after reduced heat shock. LROs are visualized using autofluorescence in blue channel. Scale bar = 20 µm. (B) Confocal fluorescence images of worms carrying an extrachromosomal array expressing HSP-90::GFP$^{int\text{-}OE}$ both in a wild-type and *cul-6(ok1614)* mutant background 1 h after reduced heat shock (left column), presented with an area of interest outlined in white (magnified in right column). Left scale bar = 20 µm, right scale bar = 5 µm. (C) Quantification of the fraction of worms showing intestinal ring-like HSP-90::GFP structures 1 hour after reduced heat shock in a wild-type and *cul-6(ok1614)* mutant background. n = 137 per strain across three independent experiments.

Application	Sequence(s)
pals-22(jy1) genotyping (*TaqI* Digest)	CCACACCTGGCACATAAAATC, GGTCTGACATAAGCCTACAAG
scav-3(ok1286) genotyping	GACAAGACTAGTCCGCCAGC, TGTGCGGCACCTTGCAAACTCA, GGTCATTGTGACCCGTAAGC
cul-6(ok1216) genotyping	GCACCATCGAATGGGACAAC, CTCACTACGGCATCAGGTGG, GGATCCCAAGTTGTACGGCA
hsf-1(sy441) genotyping	GTACCGGCACATCAAATCCA, GTGGCTTCATGCCTTCAGAT
hsf-1(sy441) sequencing	GTACCGGCACATCAAATCCA
rde-1(ne300) genotyping	AATTGCTCAGAGAATTCGCAGAA, ACAATTCCAGTTTCTTTGCTTTCTT
rde-1(ne300) sequencing	AGCGACATCTGTTTCAGCAG

Table S1. Primers used in this study. Related to STAR methods.

Strain Name	Genotype (transgene or mutant allele details)	Source	Notes	Figure Appearance
N2	wild-type	Caenorhabditis Genetics Center		1-5, S2
ERT356	*pals-22(jy1) III*	Reddy *et al*., 2017		1+D3
ERT571	*jySi42[pET499(vha-6p::GFP::cul-6::unc-54 3' UTR, unc-119(+))] II; unc-119(ed3) III*	(Panek *et al*., 2020)		3-5, S4
ERT740	*jySi46[pET688(vha-6p::GFP::cul-6(K673R)::unc-54 3' UTR, unc-119(+))] II; unc-119(ed3) III*	(Panek *et al*., 2020)		3-5, S4
RB938	*vha-12(ok821) X*	Caenorhabditis Genetics Center		1
AM994	*hsp-90control sid-1(pk3321); rmIs288(myo-2p::CFP;hsp-70p::mCherry)*	van Oosten-Hawle *et al*., 2013		2
PVH1	*sid-1(pk3321) V;rmIs288[myo-2p::CFP;hsp-70p::mCherry];pcIs001[rgef-1p::hsp-90RNAi::unc-54 3'UTR]*	van Oosten-Hawle *et al*., 2013		2
PVH2	*sid-1(pk3321) V;rmIs288;pcIs002[vha-6p::hsp-90RNAi::unc-54 3'UTR]*	van Oosten-Hawle *et al*., 2013		2
PS3551	*hsf-1(sy441) I*	Caenorhabditis Genetics Center		2, S2
ERT1006	*scav-3(ok1286) III*	Caenorhabditis Genetics Center	backcrossed into N2 from RB1227	1
ERT1236	*rmIs346[vha-6p::HSP-90::RFP]*	van Oosten-Hawle et al., 2013	backcrossed into N2 from AM986	2-5, S2, S4
ERT1226	*rmIs345[F25B3.3p::HSP-90::RFP]*	van Oosten-Hawle et al., 2013	backcrossed into N2 from AM987	2
ERT1227	*rmIs347[unc-54p::HSP-90::RFP]*	van Oosten-Hawle et al., 2013	backcrossed into N2 from AM988	2
ERT1166	*njIs11[glr-3p::GFP + ges-1p::RFP]*	Caenorhabditis Genetics Center	backcrossed into N2 from IK716	S2
ERT1167	*njIs12[glr-3p::glr-1::GFP + glr-3p::RFP + ges-1p::RFP]*	Caenorhabditis Genetics Center	backcrossed into N2 from IK718	S2
ERT1004	*pals-22(jy1) scav-3(ok1286) III*	This paper	cross between ERT356 and ERT1006	1
ERT1152	*jySi42 II; unc-119(ed3) scav-3(ok1286) III*	This paper	cross between ERT571 and ERT1006	1
ERT1046	*cul-6(ok1614) IV;rmIs346*	This paper	cross between ERT540 and ERT1236	3-5, S4
ERT1104	*jySi42 II; unc-119(ed3) III; rmIs346*	This paper	cross between ERT571 and ERT1236	3-5, S4
ERT1136	*jySi46 II; rmIs346*	This paper	cross between ERT740 and ERT1236	3-5, S4
ERT1212	*scav-3(ok1286) III; rmIs346*	This paper	cross between ERT1006 and ERT1236	3, 4
ERT1265	*frSi17[mtl-2p::rde-1 3'UTR] II; rde-1(ne300) V*	Caenorhabditis Genetics Center	backcrossed into N2 from IG1839	3
ERT1295	*frSi17[mtl-2p::rde-1 3'UTR] II; rde-1(ne300) V; rmIs346[vha-6p::HSP-90::RFP]*	This paper	cross between ERT1265 and ERT1236	3
ERT1297	*LMP-1:GFP;HSP-90::RFP*	This paper	cross between ERT1296 and ERT 1236	5
CL2070	*dvIs70[hsp-16-2p::GFP + pRF4 rol-6(su1006)]*	Caenorhabditis Genetics Center		S2
ERT1191	*hsf-1(sy4410) I; rmIs346*	This paper	cross between PS3551 and ERT1236	S2
ERT1210	*qxIs430 [scav-3::GFP + unc-76(+)]*	Caenorhabditis Genetics Center	backcrossed into N2 from XW8056	S2
ERT1237	*qxIs430;rmIs346*	This paper	cross between ERT1210 and ERT1236	S2
ERT1238	*jyEx128 [vha-6p::GFP::UBQ, cb-unc-119(+)]; unc-119(ed3)III; rmIs346*	This paper	cross between ERT261 and ERT1236	S3
ERT1239	*jyEx131 [vha-6p::GFP::UBQdeltaGG, cb-unc-119(+)]; unc-119(ed3)III; rmIs346*	This paper	cross between ERT264 and ERT1236	S3
ERT1298	*rmEx315[vha-6p::HSP-90::GFP; myo-2p::mCherry]*	van Oosten-Hawle et al., 2013	backcrossed into N2 from PVH301	S4
ERT1299	*cul-6(ok1614) IV;vha-6p::HSP-90::GFP*	This paper	cross between ERT540 and ERT1298	S4

Table S2. Strains of *C. elegans* used in this study and their appearance in figures. Related to STAR methods.
